# EzrA Contributes to the Regulation of Cell Size in *Staphylococcus aureus*

**DOI:** 10.1371/journal.pone.0027542

**Published:** 2011-11-14

**Authors:** Ana M. Jorge, Egbert Hoiczyk, João P. Gomes, Mariana G. Pinho

**Affiliations:** 1 Laboratory of Bacterial Cell Biology, Instituto de Tecnologia Química e Biológica, Universidade Nova de Lisboa, Oeiras, Portugal; 2 The W. Harry Feinstone Department of Molecular Microbiology and Immunology, Johns Hopkins Bloomberg School of Public Health, Baltimore, Maryland, United States of America; 3 National Institute of Health, Department of Infectious Diseases, Lisbon, Portugal; University of Edinburgh, United Kingdom

## Abstract

EzrA is a negative regulator of FtsZ in *Bacillus subtilis*, involved in the coordination between cell growth and cell division and in the control of the cell elongation–division cycle. We have now studied the role of the *Staphylococcus aureus* homologue of the *B. subtilis* EzrA protein and shown that it is not essential for cell viability. EzrA conditional and null mutants have an overall increase of the average cell size, compared to wild type strains. In the larger *ezrA* mutant *S. aureus* cells, cell division protein FtsZ and the cell wall synthesizing Penicillin Binding Proteins (PBPs) are not properly localized. This suggests that there may be a maximum cell diameter that allows formation of a Z-ring capable of recruiting the other components of the divisome and of driving cytokinesis. We propose that the major role of EzrA in *S. aureus* is in cell size homeostasis.

## Introduction

Cell division in bacteria is a highly regulated process during which cells undergo a series of temporally and spatially controlled events that result in the generation of two identical daughter cells [1]. In nearly all bacteria, cell division initiates with the polymerization of a tubulin-like protein, FtsZ, into a ring structure located at the future division site [1], [2]. This Z-ring serves as a scaffold for the recruitment of other proteins involved in cell division, resulting in the assembly of a multiprotein complex known as the divisome [1], [3]. Constriction of the Z-ring and concomitant synthesis of new cell wall material initiate division septum formation, a process that eventually leads to the separation and release of two daughter cells.

In *Bacillus subtilis*, a model organism for the study of cell division, the concentration of FtsZ during the cell cycle remains constant [4]. This fact implies that timing of FtsZ assembly is not regulated via oscillations in the availability of FtsZ monomers, but primarily through cell cycle-dependent variations in FtsZ polymerization [4]. In agreement with this idea, previous studies have identified various septal proteins that modulate Z-ring assembly and dynamics: FtsA [5] and ZipA [6] (present in Gammaproteobacteria), which promote Z-ring formation and anchor FtsZ to the membrane; ZapA, a small protein which enhances both the assembly and the stability of the Z-ring [7]; ZapB (present in a subset of Gammaproteobacteria), which may promote the organization of shorter FtsZ protofilaments into a functional ring [8]; SepF (present in Gram-positive bacteria and in Cyanobacteria) which forms rings required for the regular arrangement of FtsZ filaments [9], [10]; and EzrA (found in Gram-positive bacteria with low GC content), a negative regulator of Z-ring assembly [11].

EzrA (for *e*xtra *Z-*rings A) is an abundant protein with an estimated 10,000–20,000 molecules per *B. subtilis* cell, anchored to the membrane by an N-terminal transmembrane domain [12]. In *B. subtilis*, EzrA acts as an inhibitor that prevents formation of aberrant Z-rings at the poles of exponentially growing cells, ensuring the formation of only one Z-ring per cell cycle, at mid-cell [11]. The absence of EzrA lowers the critical concentration of FtsZ necessary to form a Z-ring, probably due to stabilization of the FtsZ ring [11], [13]. EzrA interacts directly with the C-terminus of FtsZ, inhibiting FtsZ assembly *in vitro* [12], [14] as this interaction not only reduces the GTP-binding affinity of FtsZ, but also increases the rate of GTP hydrolysis, both of which are unfavorable for FtsZ polymerization [15].

In *B. subtilis*, EzrA localizes to the cell membrane, where it is proposed to prevent FtsZ assembly at inappropriate locations other than mid-cell [11]. However, EzrA also localizes to the divisome at mid-cell [11], a localization which appears to be contradictory with EzrA's role as an FtsZ negative regulator. It seems that the presence of EzrA in the divisome reflects a second role of EzrA in maintaining proper FtsZ dynamics within the medial Z-ring [16] perhaps contributing to Z-ring remodeling by accelerating the disassembly of the Z-ring as cytokinesis progresses [7]. This latter role of EzrA may be important for the coordination between cell growth and cell division, as a strain encoding EzrA mutated in the conserved “QNR patch”, required for EzrA localization to the medial Z-ring (but not for EzrA inhibition of FtsZ assembly at the poles) has an increased cell length [16]. More recently, EzrA together with the newly identified divisome component GpsB, has been proposed to have a third role, related to the control of the cell elongation–division cycle of *B. subtilis*, by modulating the recruitment of Penicillin-Binding Protein 1 (PBP1) to the divisome [17]. PBPs are enzymes responsible for the last stages of peptidoglycan biosynthesis, required for cell elongation as well as for the synthesis of the division septum in rod-shaped bacteria.

Cell division in general, and the regulation of the Z-ring in particular, has been studied mostly in the rod-shaped model bacteria *Escherichia coli* and *B. subtilis*. However, lessons learned from these organisms may not always be applicable to other bacteria, namely to bacteria with different morphologies. One interesting alternative model to study cell division is *Staphylococcus aureus* because it has spherical cells, and therefore, it is likely to have different mechanisms for placement of the division septum. Moreover, the bacterial cell division machinery constitutes a good potential target for the development of new antibiotics [18] and therefore it is important to understand this process in clinically relevant bacteria. *S. aureus* is a leading nosocomial pathogen due to its extraordinary capacity to acquire resistance to virtually all classes of available antibiotics. In fact, Methicillin-Resistant *S. aureus* strains (MRSA) currently cause more deaths in the USA than HIV/AIDS and tuberculosis combined [19].

In this work we studied the first steps of cell division in *S. aureus*. In particular, we focused upon the role of EzrA, which had been identified in a transposon-mediated differential hybridisation screen as a putative essential gene in *S. aureus* [20]. While this manuscript was being prepared, Steele and colleagues also reported that EzrA was essential in *S.aureus* by expressing it under the control of an inducible promoter [21]. Intriguingly, this result indicated that, contrary to what is observed in *B. subtilis*, EzrA could play an essential role in *S. aureus*, pointing to important differences between these two bacterial species. Using various approaches, we now show that EzrA is not essential in *S. aureus* but is required for maintaining correct cell size. In the absence of EzrA, there is a significant increase of the average diameter of *S. aureus* cells. Moreover, larger cells were found to have delocalized FtsZ and PBPs, suggesting that there may be a maximum cell diameter for proper assembly of the divisome in *S. aureus*.

## Results

### EzrA is not essential in *Staphylococcus aureus*

Before we started the studies reported here, the gene encoding the *S. aureus* homologue of the *B. subtilis* cell division protein EzrA had been identified as an essential gene by Transposon-Mediated Differential Hybridisation screening [20]. As *B. subtilis* EzrA is not essential for survival, we hypothesized that its function could be more relevant in *S. aureus*. We therefore constructed conditional *ezrA* mutants, in order to confirm its essentiality and to study its role in cell division of Gram-positive cocci.

**Figure 1:**
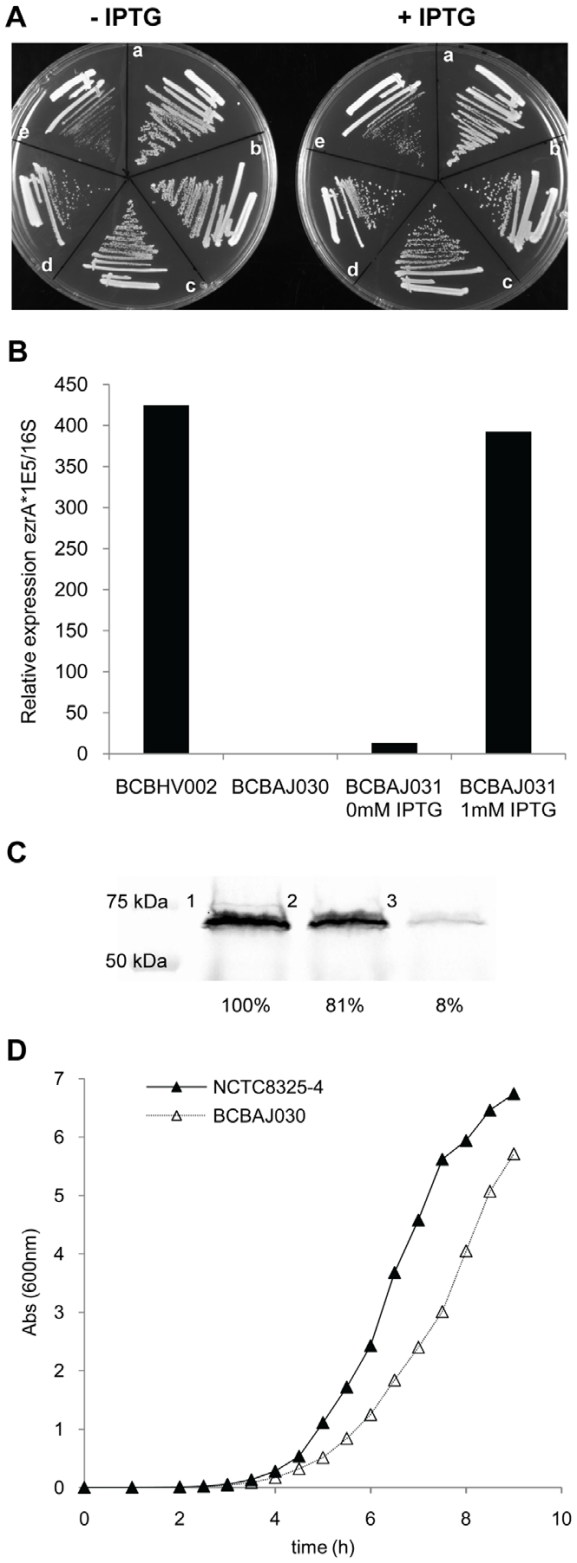
EzrA is not essential in *S. aureus*. (A) Bacterial strains with *ezrA* under the control of inducible P*_spac_* promoter, grown on solid medium (TSA) supplemented or not supplemented with 1 mM IPTG. (a) BCBAJ036 (RN4220 background); (b) BCBAJ031 (NCTC8325-4 background); (c) BCBAJ034 (SH1000 background); (d) BCBAJ035 (Newman background); (e) BCBAJ019 (COL background). (B) Expression of *ezrA* transcript in control strain BCBHV002 (NCTC8325-4, *lacI*) and in the inducible strain BCBAJ031 (NCTC8325-4 *ezrA*:: P*_spac_* -*ezrA*, *lacI*), grown in the presence and in the absence of IPTG, quantified by RT-PCR and normalized using 16sRNA. (C) Analysis, by SDS-PAGE, of cell lysates of strains BCBAJ012 (COL *ezrA*::*ezrA-mCherry*, lane 1) and BCBAJ024 (COL *ezrA*::*ezrA-mCherry*, containing pBCBAJ006 plasmid expressing *ezrA* antisense RNA) grown in the presence of glucose 0.2% (w/v) to repress antisense RNA transcription (lane 2), or in the presence of xylose 2% (w/v) to induce antisense RNA transcription (lane 3). Bands correspond to fluorescence of EzrA-mCherry detected using a FUJI FLA5100 reader. Integrated density for each band is shown below. (D) Growth curve of wild type strain NCTC8325-4 and null mutant BCBAJ030 (NCTC8325-4 *ΔezrA*) in liquid medium (TSB).

As a first approach we constructed strain BCBAJ031 in which the *ezrA* gene was placed under the control of the IPTG-inducible, LacI-repressible P*_spac_* promoter. For that purpose, the *ezrA* ribosomal binding site (RBS) together with the initial 533 bp of the *ezrA* gene were cloned downstream of the P*_spac_* promoter in the pMUTIN4 vector, which was then integrated into the *ezrA* locus of the *S. aureus* chromosome of strain NCTC8325-4. The plasmid pMGPII, containing a second copy of the *lacI* gene, was also introduced into this strain to enhance repression of *ezrA* transcription. The resulting strain BCBAJ031 was able to grow in the absence of the inducer IPTG (Fig. 1A) indicating that EzrA was not essential. EzrA was also placed under the control of the inducible P*_spac_* promoter in the background of strains RN4220 (BCBAJ036), COL (BCBAJ019), SH1000 (BCBAJ034) and Newman (BCBAJ035) which, similarly to BCBAJ031, were able to grow in the absence of IPTG both in solid (Fig. 1A) and in liquid medium (data not shown). To confirm that *ezrA* transcription was indeed repressed in the absence of inducer, we used quantitative real time PCR (RT-PCR) to quantify the amount of *ezrA* transcript in control strain BCBHV002 (NCTC8325-4 pMGPII) and in the *ezrA* inducible BCBAJ031 strain, grown in the presence and in the absence of IPTG. In the absence of inducer, the amount of *ezrA* transcript was 33 times lower than in the parental strain BCBHV002 while addition of IPTG (1 mM) led to recovery of nearly wild type levels of *ezrA* mRNA (Fig. 1B).

As a second approach to generate a conditional *ezrA* mutant, we constructed the replicative plasmid pBCBAJ006, encoding *ezrA* antisense RNA under the control of the xylose-inducible, glucose-repressible promoter pT5X. This plasmid was introduced into strains RN4220, SH1000, NCTC8325-4, Newman and COL. The empty pEPSA5 vector was introduced into the same strains and used as negative control. Growth of the resulting strains in the presence of 2% xylose (to induce antisense RNA expression and therefore repress *ezrA* expression) did not result in growth halt (data not shown), again indicating that EzrA is not essential in *S. aureus*. Depletion of EzrA due to *ezrA* antisense RNA expression was confirmed using strain BCBAJ024 expressing an EzrA-mCherry fusion protein from its native chromosomal locus. Quantification of EzrA-mCherry fluorescence in this strain grown in the presence of xylose or glucose showed that there was a 92% reduction in the amount of EzrA-mCherry protein produced when the *ezrA* antisense RNA was expressed (Fig. 1C).

Finally, in order to show that *S. aureus* cells were able to grow in the complete absence of EzrA, we constructed null mutants in the background of five *S. aureus* strains RN4220, NCTC8325-4, SH1000, Newman and COL by deleting the entire *ezrA* coding sequence, with the exception of its start and stop codons. For that purpose, up and downstream regions of the *ezrA* gene were cloned into the thermosensitive pMAD vector, which was then integrated into the *S. aureus* chromosome at 43°C. Growth in the absence of antibiotic selection allowed excision of the plasmid, which resulted in either wild type or null mutant strains. PCR screening of colonies in which plasmid excision had occurred resulted in the identification of an average of 46% of null mutants (the exact value depends on the background strain used), showing that EzrA is not essential in *S. aureus*. Growth curves of NCTC8325-4 and its *ezrA* null mutant BCBAJ030 show that absence of EzrA results in a small growth defect (Fig. 1D)

### Absence of EzrA disturbs cell size homeostasis

**Figure 2:**
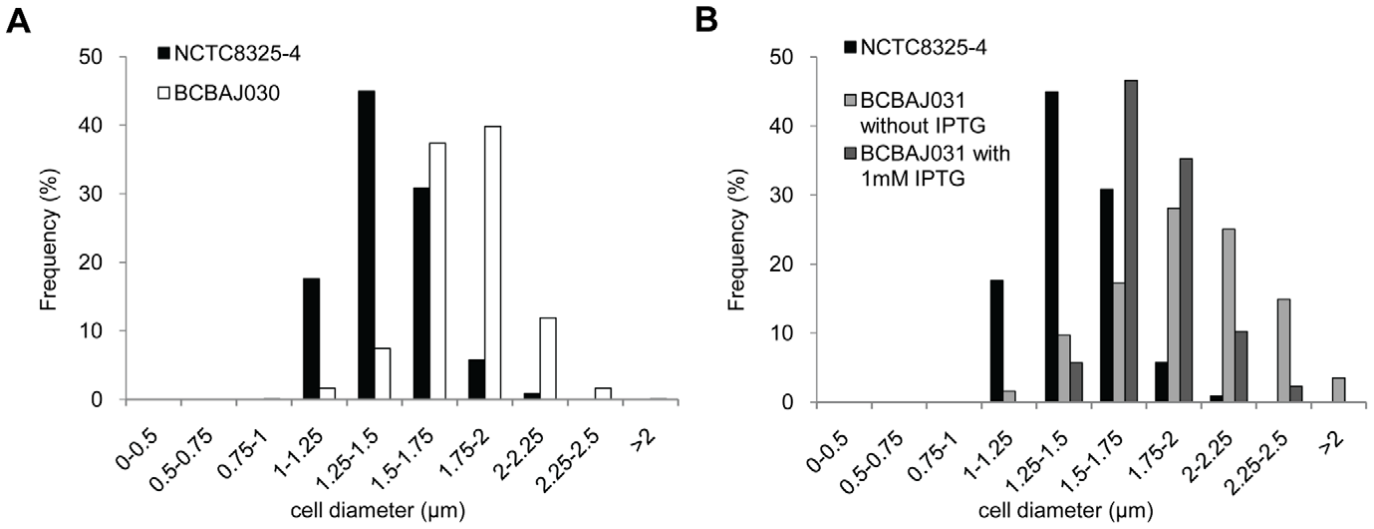
Absence of EzrA in *S. aureus* leads to an increase in cell diameter. Phase contrast images were used to measure the diameter of 600 to 1000 cells of each strain: wild type NCTC8325-4, null mutant BCBAJ030 (NCTC8325-4 *ΔezrA*), and *ezrA* conditional mutant BCBAJ031 (NCTC8325-4 *ezrA*:: P*_spac_ -ezrA*, *lacI*) grown in the presence and in the absence of 1 mM of IPTG inducer.

In order to study the role of EzrA in *S. aureus*, we first examined the EzrA mutant strains by phase microscopy. Strains BCBAJ031 (in which *ezrA* is under the control of P*_spac_* in the NCTC8325-4 background and therefore is not expressed in the absence of IPTG), or BCBAJ030 (NCTC8325-4Δ*ezrA*) showed an increased heterogeneity in cell size when compared to the corresponding controls. We therefore measured the diameter of around 1000 cells from each strain showing that lack of EzrA resulted in an overall increase of the average cell size (Fig. 2). We repeated our measurements using EzrA inducible and null mutant cells in RN4220, SH1000, Newman and COL backgrounds and again we observed an increase in average cell size of cells grown in the absence of EzrA when compared to the parental strains (Table S1).

While in rod-shaped *B. subtilis* inhibition of cell division results in longer cells [22], in spherical *S. aureus* cells inhibition of cell division, for example through depletion of FtsZ, results in larger cells which can have up to twice the diameter of a normal cell [23]. It is therefore possible that the larger size of *S. aureus* cells lacking EzrA results from a delay in cell division due to lack of proper coordination between cell growth and cell division.

### EzrA localizes to the division septum in *S. aureus* dividing cells

**Figure 3:**
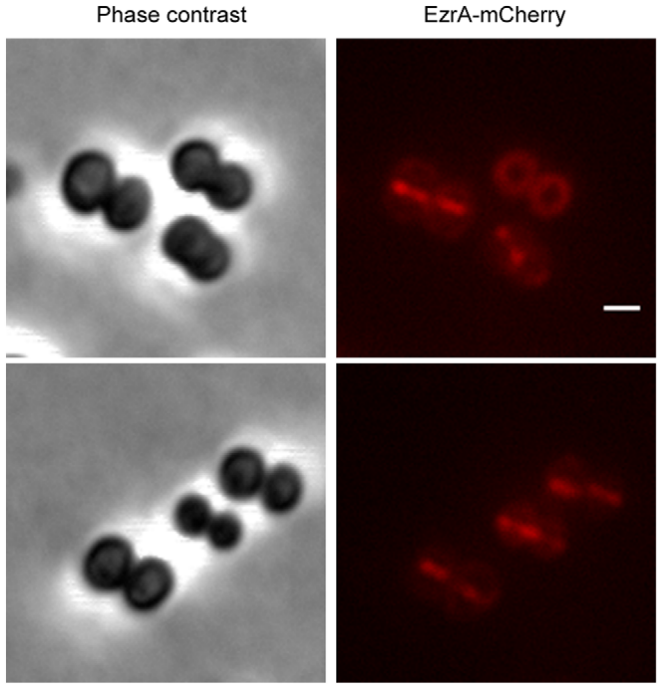
EzrA-mCherry localizes to the division septa of *S. aureus*. Phase contrast (left panels) and fluorescence (right panels) images of BCBAJ025 cells (NCTC8325-4 *ezrA*::*ezrA-mCherry*) expressing EzrA-mCherry fusion. Scale bar 1 µm.

In *B. subtilis* the proposed role of EzrA in the coordination between cell growth and cell division is dependent on its mid-cell localization. However, EzrA also localizes to the lateral membrane, which is required for its role in preventing FtsZ assembly at inappropriate locations different from the mid-cell. We have recently reported that EzrA-CFP localized to the division septa in *S. aureus* [24]. Analysis of EzrA localization pattern in the strain BCBAJ025 expressing EzrA-mCherry showed that EzrA was absent from the cell membrane that surrounds the staphylococcal cells undergoing division (Fig. 3). It therefore became interesting to determine if EzrA had any role in preventing the formation of extra Z-rings in *S. aureus*.

### EzrA is required for correct formation of the FtsZ ring in *S. aureus*

**Figure 4:**
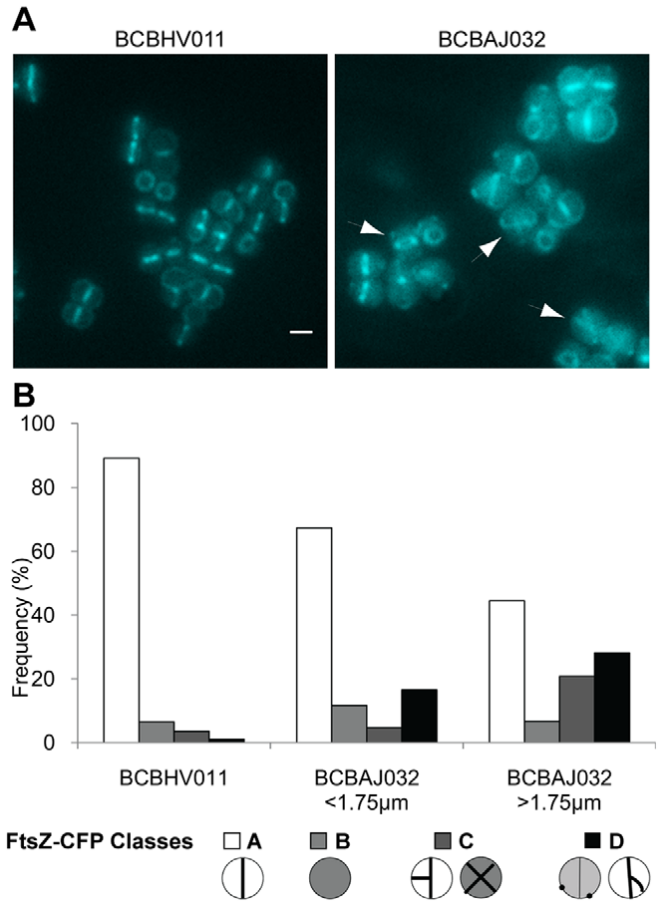
FtsZ mislocalizes in large *S. aureus* cells depleted of EzrA. (A) Fluorescence images of parental strain BCBHV011 (NCTC8325-4 *spa*::P*_spac_ftsz-cfp*) and *ezrA* null mutant BCBAJ032 (NCTC8325-4 *ΔezrA spa*::P*_spac_ftsz-cfp*) showing cells expressing FtsZCFP fusion protein. Arrows point to irregular localizations of FtsZ. Scale bar 1 µm. (B) Frequency of cells with septal localization of FtsZ-CFP (class A), fluorescence all over the cytoplasm (class B), double septa (class C) and irregular localizations of FtsZCFP (class D), for BCBHV011 (parental strain; NCTC8325-4 *spa*::P*_spac_ftsz-cfp*) and BCBAJ032 (NCTC8325-4 *ΔezrA spa*::P*_spac_ftsz-cfp*). Cells of null mutant strain BCBAJ032 were divided in two classes - cells with a diameter smaller or larger than 1.75 µm – which were analyzed separately.

**Figure 5:**
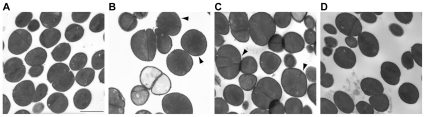
Absence of EzrA leads to abnormalities in septum formation. Electron microscopy images of wild type strain NCTC8325-3 (A), *ezrA* null mutant strain BCBAJ030 (NCTC8325-4 *ΔezrA*) (B), *ezrA* conditional mutant BCBAJ031 (NCTC8325-4 *ezrA*:: P*_spac_ -ezrA*, *lacI*) grown in the absence of IPTG inducer (C) and in the presence of 1 mM IPTG inducer (D). Arrowheads point to cells in which extra septa started to form. Scale bar 1 µm.

To study the localization of FtsZ in EzrA-depleted *S. aureus* cells we expressed FtsZ-CFP from the ectopic *spa* locus, under control of the IPTG-inducible P*_spac_* promoter in the NCTC8325-4 Δ*ezrA* strain (BCBAJ032). We observed mislocalization of FtsZ in approximately 25% of the cells in the Δ*ezrA* mutant compared to 4.5% in NCTC8325-4 cells expressing FtsZ-CFP (BCBHV011) (Fig. 4). FtsZ-CFP mislocalization patterns included protein present as dots around the membrane, protein delocalized all over the cell surface or the formation of incomplete, often asymmetrical, Z-rings (Fig. 4), as well as the formation of multiple complete FtsZ rings in a single cell in 7% of the cells. Interestingly, mislocalization of FtsZ-CFP was more pronounced in the subpopulation of very large EzrA mutant cells (diameter >1.75 µm). Results obtained by immunofluorescence using an anti-FtsZ polyclonal antibody were in agreement with these results (data not shown). Electron microscopy also confirmed the formation of extra incomplete septa in the *ezrA* inducible mutant BCBAJ031 grown in the absence of IPTG and in the null mutant BCBAJ030 (Fig. 5 C and B).

### Absence of EzrA leads to mislocalization of cell wall synthetic proteins

**Figure 6:**
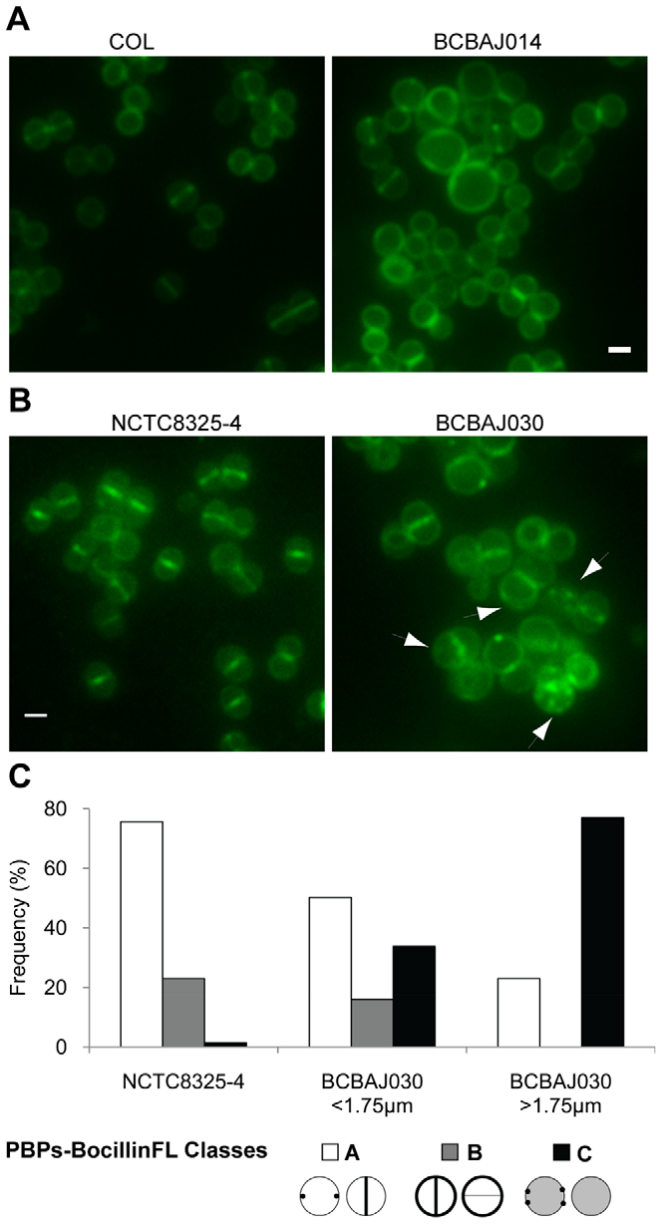
Penicillin Binding Proteins (PBPs) mislocalize in large *S. aureus* cells depleted of EzrA. (A) COL (parental strain) and BCBAJ014 (COL *ΔezrA*) cells labeled with Vancomycin-FL to visualize sites of incorporation of new cell wall. (B) NCTC8325-4 (parental strain) and BCBAJ030 (NCTC8325-4 *ΔezrA*) cells labeled with Bocillin-FL. Arrows point to cells with irregular localization of PBPs. Scale bar 1 µm. (C) Frequency of cells showing septal localization of PBPs (class A), localization around the membrane (class B) or irregular localization of PBPs (class C) for NCTC8325-4 and BCBAJ030 cells. The later were divided in two classes - cells with a diameter smaller or larger than 1.75 µm – which were analyzed separately.

As stated above, lack of EzrA resulted in an increase of the average *S. aureus* cell size. Approximately 72% of cells had a cell diameter larger than 1.75 µm in the inducible mutant BCBAJ031 or 54% in the null mutant BCBAJ030, while only 7% of wild type NCTC8325-4 cells were this large. Given that *S. aureus* synthesizes cell wall mainly, if not only, at the septum [23] we wondered how cells were able to grow to such large sizes. To investigate this question, we tested if depletion of EzrA affected the localization of cell wall synthesis and cell wall synthetic proteins (PBPs) in *S. aureus*. For that purpose we labeled nascent cell wall using a fluorescent derivative of vancomycin (Van-FL) which binds the terminal D-Ala-D-Ala residues of the peptidoglycan muropeptides [23]. Cells of strain COL (parental strain) and BCBAJ014 (COL *ΔezrA*) were grown in the presence of an excess of D-serine, which leads to the replacement of the carboxyl-terminal D-alanine residue of the peptidoglycan precursor by a D-serine residue. Cells were then changed to growth medium without D-serine to allow incorporation of peptidoglycan precursors with D-Ala-D-Ala termini into the nascent cell wall. Labeling of the resulting cells with Van-FL showed that in larger cells from strain BCBAJ014, cell wall synthesis occurred around the cell periphery, in a dispersed manner (Fig. 6A). To study the localization of cell wall synthetic enzymes, we used a fluorescent derivative of penicillin, Bocillin-FL, which binds PBPs and therefore can be used to determine the localization of these enzymes. PBPs were labeled in NCTC8325-4 (parental strain) and BCBAJ030 (NCTC8325-4 *ΔezrA*) cells using Bocillin-FL at concentrations below the MIC (minimum inhibitory concentration), for 5 minutes, to minimize its effect on the metabolism of cell wall synthesis. Under these conditions, 48% of the total BCBAJ030 cells analyzed (n = 767) kept a normal mid cell localization of these proteins (class A in Fig. 6C), while 38% had displacement and irregular distribution of PBPs (class C in Fig. 6C). Furthermore, when these results were correlated with the size of each cell, we noticed that 77% of cells with a diameter over 1.75 µm had a delocalized Bocillin-FL signal, indicating that the cell wall synthetic machinery was delocalized in these cells (Fig. 6C).

**Figure 7:**
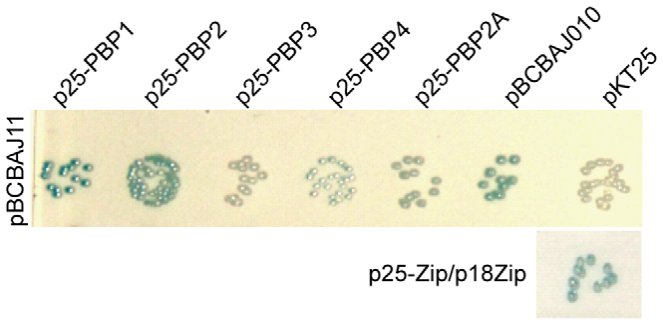
EzrA interacts with PBPs in a Bacterial Two Hybrid assay. Plasmid pBCBAJ011 (encoding EzrA-T18) was co-transformed with plasmid p25PBP1, p25PBP2, p25PBP3, p25PBP4, p25PBP2A, pBCBAJ010 (encoding EzrA-T25) and pKT25 (empty vector, negative control) and a 10^−2^ dilution of each resulting culture was plated. Plasmids p25Zip and p18Zip were used as positive control. Formation of blue colonies indicates putative interaction between cloned proteins, namely between EzrA and itself, PBP1 and PBP2.

In *B. subtilis*, EzrA has recently been proposed to have a role in the recruitment of PBP1 to the septum [17]. PBP1 is the major transglycosylase/transpeptidase bifunctional PBP in *B. subtilis*, similarly to PBP2 in *S. aureus*. To investigate if EzrA interacted with PBPs in *S. aureus*, we performed a bacterial-two hybrid assay using the BATCH system based on a cyclic AMP signaling cascade in *E. coli* [25]. This indicated that EzrA can interact with itself, with PBP1 and PBP2 (Fig. 7).

**Figure 8:**
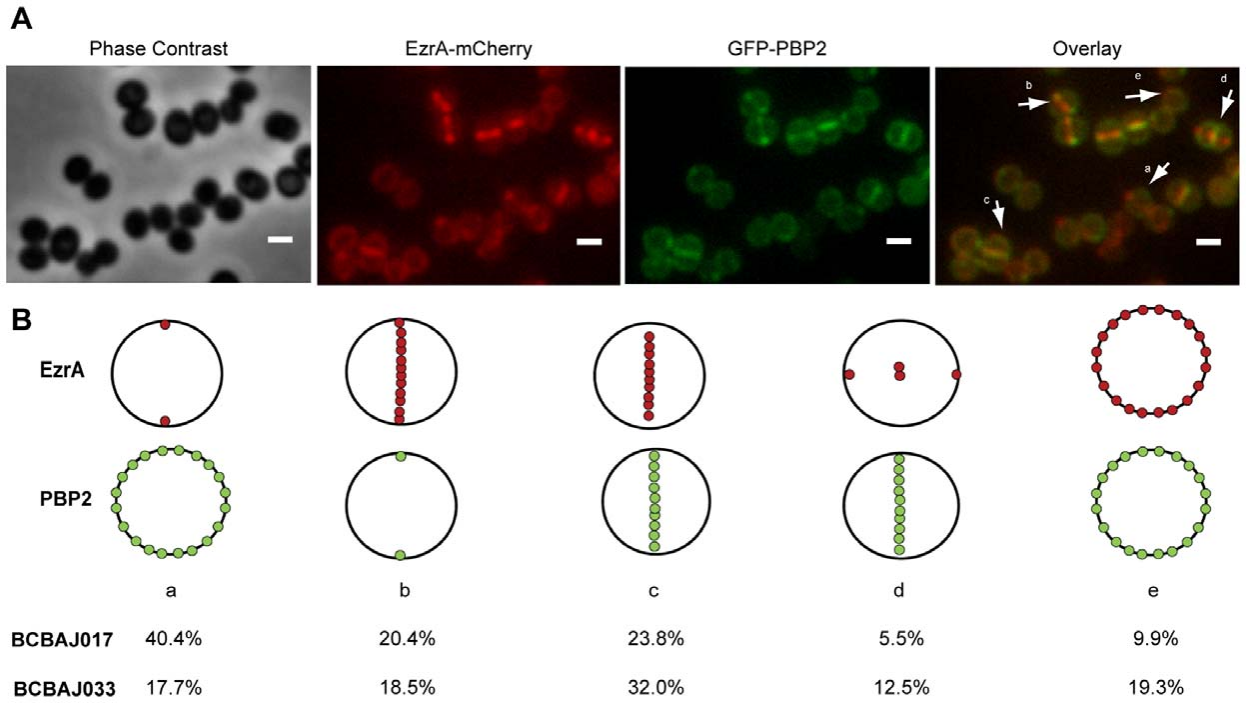
EzrA-mCherry and GFP-PBP2 do not colocalize during the entire cell cycle. (A) From left to right: Phase contrast, EzrA-mCherry fluorescence, GFP-PBP2 fluorescence and overlay of EzrA-mCherry and GFP-PBP2 fluorescence images of strain BCBAJ017 expressing, simultaneously, EzrA-mCherry and GFP-PBP2 in the COL background. Arrows show cells representative for each class (see below). (B) Localization of EzrA-mCherry and GFP-PBP2 was analyzed in 1420 cells for strain BCBAJ017 (COL background) and 802 cells for strain BCBAJ033 (NCTC8325-4 background) which were assigned to the following classes: (a) EzrA-mCherry localized in two spots, corresponding to the beginning of septa formation while GFP-PBP2 is not yet present at mid cell; (b) EzrA-mCherry is present across the entire septum while GFP-PBP2 is present as two dots, corresponding to a ring around the septum; (c) both proteins co-localize at the septum; (d) EzrA-mCherry is starting to localize to the next division site and GFP-PBP2 is still present in the previous septum; (e) both proteins are dispersed at the cell membrane. Red dots represent EzrA-mCherry and green dots GFP-PBP2. Frequency of cells in each class was calculated for BCBAJ017 (COL *ezrA::ezrA-mCherry, pbp2::gfp-pbp2*) and BCBAJ033 (NCTC8325-4 *ezrA::ezrA-mCherry, pbp2::gfp-pbp2*).

However, if EzrA does indeed interact with PBPs in *S. aureus*, these interactions (at least with PBP2) may occur only during a specific time during the cell cycle, as EzrA and PBP2 are not recruited to the septum at the same time (Fig. 8). This was seen in strain BCBAJ033 (NCTC8325-4 background) and BCBAJ017 (COL background) expressing both *ezrA-mCherry* and *gfp-pbp2* fusions from their respective native loci. Although both EzrA and PBP2 were visible at mid cell, as expected, co-localization occurred in 51% (n = 802) or 34% (n = 1420) of the cells for NCTC8325-4 and COL backgrounds, respectively (Fig. 8B). In the remaining cells the two proteins did not colocalize, as EzrA was seen in early forming septa (visible as two spots at the membrane) while PBP2 was not yet present at mid cell (class a, Fig. 8B); EzrA was found localized over the entire septum (seen as a line across the cell) while PBP2 was still present in a ring around the cell (visible as two spots at the membrane) (class b, Fig. 8B); or, when cells started to prepare the next round of division, EzrA could already be found at the next division site, whereas PBP2 was still present at the mid cell of the two daughter cells which were splitting (class d, Fig. 8B).

## Discussion

In order to study the role of EzrA in *S. aureus*, we have used three different approaches to deplete *S. aureus* cells of EzrA: placing the gene under the control of the inducible promoter P*_spac_*, expressing antisense RNA and constructing null mutants in which the complete *ezrA* gene was deleted. This was performed in the background of five different strains – RN4220, NCTC8325-4, SH1000, Newman and COL, generating a total of 15 strains which are able to grow in the absence of EzrA, indicating that EzrA is not an essential protein in *S. aureus*. These results are not in agreement with those recently reported by Steele and colleagues, using a SH1000 strain expressing *ezrA* under the control of the same P*_spac_* inducible promoter [21]. One hypothesis for the discrepancy between our results and those from Steele and colleagues would be the presence of suppressor mutations in the strains reported here, which would allow them to grow in the absence of EzrA. However, we think that is unlikely because null mutants were obtained at rates up to 70%. If a suppressor mutation was required for the viability of the null mutants, we would expect a strong predominance of wild type colonies during the excision of the plasmid pBCBAJ003 used to construct the null mutants (this excision can result either in wild type or Δ*ezrA* cells, see materials and methods). A second reason to discard the hypothesis of suppressor mutations is that is does not explain the viability of the *ezrA* inducible mutants we have constructed. These strains, in which *ezrA* expression in under the control of IPTG-inducible P*_spac_* promoter, were maintained in the constant presence of high (1 mM) concentrations of IPTG, i.e., in the constant presence of EzrA, except during the depletion experiments. Therefore these conditions should not favor selection of suppressor mutations for EzrA absence.

One possible explanation for the lack of growth, in the absence of inducer, of the *ezrA* inducible strain VF79 reported by Steele and colleagues could be the presence of a mutation synthetic lethal with ezrA, that could have been transduced from the background of RN4220 (a mutagenized strain) into SH1000 during the construction of VF79. This hypothesis is based on the fact that mutations in at least five genes have been shown to be synthetic lethal with the absence of *ezrA* in *B. subtilis: zapA* [7], *gpsB* [17], *sepF* [9], *noc* [26] and *ftsL* [27].

When EzrA was initially described in *B. subtilis*, the first obvious phenotype caused by lack of this protein was the formation of extra Z-rings near the poles [11]. However, later studies by Haeusser and colleagues suggested that EzrA has a second function in contributing to the maintenance of proper FtsZ assembly dynamics in the medial Z-ring [16]. This function would render the Z-ring sensitive to factors responsible for coordinating cell growth and cell division. As a consequence, impairing EzrA's ability to localize to the medial Z-ring (without impairing its ability to inhibit FtsZ assembly at the cell poles), resulted in cells with stabilized FtsZ assembly at midcell, which were significantly longer than wild type cells [16]. Importantly, these two functions of EzrA – preventing Z-ring formation at the cell poles and coordinating cell growth and cell division – are directly related to the two localization patterns observed for EzrA in *B. subtilis* cells – at the membrane and at the nascent septal site, respectively.

The most obvious phenotype in all of the *ezrA* mutants we have constructed was a difference in cell size, with *ezrA* mutants having on average larger cells than wild type strains. Given that lack of EzrA results in the presence of extra Z-rings in *B. subtilis* [11], we looked for the formation of additional Z-rings in *S. aureus* strains depleted of EzrA and expressing a CFP fluorescent derivative of FtsZ. Although we did observe mislocalization patterns for FtsZ in approximately one quarter of the cells analyzed, two complete Z-rings were found in less than 7% of the cells. This is in sharp contrast with *B. subtilis ezrA* mutants in which 54% of the cells have more than one FtsZ ring [11]. We have also observed that EzrA localizes to the division site in cells undergoing division, and is essentially absent from the cell membrane in these cells. Putting this information together, we propose that the major role for EzrA in *S. aureus* is to maintain proper FtsZ assembly dynamics at mid cell, contributing to the coordination of cell growth and cell division, and not to prevent the assembly of Z-rings at inappropriate locations. One hypothesis for the role of EzrA at mid cell in *B. subtilis* is that it contributes to Z-ring remodeling by accelerating the disassembly of the Z-ring as cytokinesis progresses [7]. Lack of EzrA therefore leads to stabilized Z-rings, which may exist for longer periods during the bacterial cell cycle. In *S. aureus*, the septum is the only place where cell wall synthesis occurs [23]. Therefore, it is conceivable that in the absence of EzrA, there is more time to synthesize cell wall at the septum and therefore additional cell wall material may be incorporated into the septum. Splitting of the staphylococcal division septum generates one hemisphere of each new daughter cell and, in principle, expansion of the flat septum to generate the inflated hemispheres could occur by limited cell hydrolysis only [28]. Therefore it is possible that a septum containing more cell wall material would lead to larger hemispheres in the daughter cells, and consequently to larger daughter cells. These cells may be able to further increase in size due to dispersed cell wall synthesis around the periphery of the EzrA mutant cells. These mechanisms could explain the larger cell sizes that we have observed in the *S. aureus ezrA* mutants.

Approximately 71% and 54% of EzrA inducible and null NCTC8325-4 mutant cells have diameters over 1.75 µm. Both FtsZ and PBPs are mostly delocalized in these large cells, raising the possibility that in the absence of EzrA, FtsZ dynamics is altered so that the Z-ring can no longer be assembled/maintained. An alternative hypothesis would be the existence of a maximum cell diameter in *S. aureus* which allowed the formation of a productive Z-ring, capable of recruiting the other protein components of the divisome and of driving cytokinesis. Above that maximum diameter, a functional Z-ring would not be stable or would not be able to form and consequently, proteins such as cell wall synthetic enzymes would not be recruited to the divisome and/or would become delocalized. This hypothesis is in agreement with previous observations made in a *S. aureus* mutant in which FtsZ was placed under the control of the IPTG-inducible P*_spac_* promoter. FtsZ-depleted cells increased in size up to twice their initial diameter [23]. This increase in size was not reversible even when IPTG was added to restore FtsZ production, indicating that a Z-ring cannot be formed in staphylococcal cells with very large diameters [23].

Lastly, we have obtained some evidence that EzrA may be part of a protein complex that includes PBPs, as EzrA is able to interact with PBP1 and PBP2 in a bacterial two-hybrid assay. These results are in agreement with the most recent role attributed to EzrA in *B. subtilis*, in promoting the recruitment of the major transglycosylase/transpeptidase PBP1 to the division site. However, if an interaction between EzrA and PBP2 (the major transglycosylase/transpeptidase of *S. aureus*) does occur in live staphylococcal cells, it is probably temporary, as although EzrA and PBP2 colocalize at the septum during certain stages of the cell cycle (see Fig. 8), microscopy images indicate that EzrA is recruited to the division septum before PBP2.

In summary, our work suggests that the major role of EzrA in *S. aureus* is to contribute to the coordination of cell growth and cell division and consequently to the maintenance of the correct size of the staphylococcal cells. Furthermore, our work reinforces the importance of studying the role of conserved cell division proteins in different model organisms, namely in organisms with different morphologies.

## Materials and Methods

### Bacterial strains, plasmids and growth conditions

All plasmids and strains used in this study are listed in Tables 1 and 2. The sequences of the primers used are listed in Table 3. *E. coli* strain DH5α was grown on Luria-Bertani agar (LA) or in Luria–Bertani broth (LB, Difco), supplemented with ampicillin (100 µg/ml) as required. *S. aureus* strains were grown on tryptic soy agar (TSA, Difco) at 37°C or in tryptic soy broth (TSB, Difco) at 37°C with aeration, unless otherwise stated. The medium was supplemented, when necessary, with 10 µg/ml of erythromycin (Sigma), 100 µg/ml of 5-bromo-4-chloro-3-indolyl-β-D-galactopyranoside (X-Gal, Apollo Scientific) or 0.1 mM (for induction of FtsZ-CFP expression) or 1 mM (for induction of EzrA expression) of isopropyl-β-D-thiogalactopyranoside (IPTG, Apollo Scientific). Conditional mutants were always grown in the presence of 1 mM IPTG except during depletion assays. Growth was monitored by the increase in optical density at 600 nm (OD_600 nm_).

**Table 1:** Plasmids used in this study.

Plasmids	Relevant characteristics	Source or Reference
pMAD	*E. coli* – *S. aureus* shuttle vector with a thermosensitive origin of replication for Gram-positive bacteria; Amp^r^, Erm^r^, *lacZ*	[35]
pEPSA5	*E. coli*–*S. aureus* shuttle vector; replicative in *S. aureus*; Amp^r^, Cm^r^; Xylose-inducible pT5X promoter, XylR	[34]
pMUTIN4	*E. coli*–*S. aureus* shuttle vector; integrative in *S. aureus*; Amp^r^, Erm^r^; IPTG-inducible P*_spac_* promoter	[32]
pMGPII	*S. aureus* replicative plasmid containing *lacI* gene, Amp^r^, Cm^r^	[33]
pBCB4-ChE	*S. aureus* integrative vector for N- and C-termini mCherry fusions, Amp^r^, Erm^r^	[24]
pBCBHV003	pMAD derivative plasmid with *ftsZ-cfp* cloned downstream of P*_spac_* promoter; Amp^r^, Erm^r^	[36]
pBCBAJ001	pMAD containing the 3′ end and the downstream region of *ezrA*, Amp^r^, Erm^r^	This study
pBCBAJ002	pBCBAJ001 with *mCherry* cloned downstream of *ezrA*, Amp^r^, Erm^r^	This study
pBCBAJ003	pMAD with up and downstream sequence of *ezrA* gene	This study
pBCBAJ006	pEPSA with an inverted 3′ sequence of *ezrA* gene for antisense RNA expression	This study
pBCBAJ009	pMUTIN4 with 5′end of *ezrA* including RBS cloned downstream of P*_spac_* promoter; Amp^r^, Erm^r^	This study
pUT18	BTH plasmid, cyaAT18, Amp^r^	[25]
pKNT25	BTH plasmid, cyaAT25, Kan^r^	[25]
pKT25	BTH plasmid, cyaAT25, Kan^r^	[25]
p18Zip	BTH control plasmid, Amp^r^	[25]
p25Zip	BTH control plasmid, Kan^r^	[25]
p25PBP1	pKT25 with *cyaAT25-pbp1* fusion, Kan^r^	[38]
p25PBP2	pKT25 with *cyaAT25-pbp2* fusion, Kan^r^	[38]
p25PBP3	pKT25 with *cyaAT25-pbpC* fusion, Kan^r^	[38]
p25PBP4	pKNT25 with *pbpD-cyaAT25* fusion, Kan^r^	[40]
p25PBP2A	pKT25 with *cyaAT25-mecA*fusion, Kan^r^	[38]
pBCBAJ010	pKNT25 with *ezrA-cyaAT25* fusion, Kan^r^	This study
pBCBAJ011	pUT18 with *ezrA-cyaAT18* fusion, Amp^r^	This study

**Table 2:** Strains used in this study.

Strains	Relevant characteristics	Source or Reference
*E. coli*		
DH5α	*recA endA*1 *gyr*A96 *thi*-1 *hsdR*17 *sup*E44 *relA*1 Φ80 d*lac*Z ΔM15	Gibco-BRL
BTH101	Reporter strain for BTH system, *cya* ^−^	[25]
*S. aureus*		
RN4220	Restriction deficient derivative of NCTC8325-4	R. Novick
NCTC8325-4	MSSA strain	R. Novick
SH1000	Functional *rsbU* ^+^ derivative of NCTC8325-4	[41]
Newman	MSSA strain	[42]
COL	MRSA strain, Tet^s^	RU collection
RNpPBP2-31	RN4220 with *gfp-pbp2* fusion at the *pbp2* locus, Erm^r^	[37]
BCBHV002	NCTC8325-4 transformed with pMGPII; Cm^r^	[36]
BCBHV011	NCTC8325-4 Δ*spa*::P_spac_-*ftsZ–cfp-lacI lacI* ^mc^; Cm^r^	
BCBAJ001	RN4220 transformed with replicative pMGPII which contains *lacI* gene, Cm^r^	This study
BCBAJ002	RN4220 transformed with pBCBAJ003, Erm^r^	This study
BCBAJ004	RN4220Δ*ezrA*	This study
BCBAJ005	RN4220 transformed with pEPSA5 vector, Cm^r^	This study
BCBAJ006	RN4220 transformed with pBCBAJ006 expressing *ezrA* antisenseRNA under the control of pT5X, Cm^r^	This study
BCBAJ010	COL transformed with pMGPII which contains *lacI* gene, Cm^r^	This study
BCBAJ011	COL transformed with replicative pBCBAJ003, Erm^r^	This study
BCBAJ012	COL *ezrA::ezrA-mCherry*	This study
BCBAJ013	COL transformed with pBCBAJ002, Erm^r^	This study
BCBAJ014	COLΔ*ezrA*	This study
BCBAJ015	COL transformed with pEPSA5 vector, Cm^r^	This study
BCBAJ016	COL transformed with pBCBAJ006 expressing *ezrA* antisense RNA under the control of pT5X, Cm^r^	This study
BCBAJ017	BCBAJ012 (COL *ezrA*::*ezrA-mCherry*) with *gfp-pbp2* fusion at the *pbp2* locus, Erm^r^	This study
BCBAJ019	COL with pBCBAJ009 integrated at *ezrA* locus expressing *ezrA* under the control of P*_spac_* and replicative pMGPII; Erm^r^, Cm^r^, *lacI*	This study
BCBAJ023	BCBAJ012 (COL *ezrA*::*ezrA-mCherry*) transformed with pEPSA5, Cm^r^	This study
BCBAJ024	BCBAJ012 (COL *ezrA*::*ezrA-mCherry*) transformed with pBCBAJ006 expressing *ezrA* antisense RNA under the control of pT5X, Cm^r^	This study
BCBAJ025	NCTC8325-4 *ezrA::ezrA-mCherry*	This study
BCBAJ028	RN4220 transformed with pBCBAJ002, Erm^r^	This study
BCBAJ030	NCTC8325-4Δ*ezrA*	This study
BCBAJ031	NCTC8325-4 with pBCBAJ009 integrated at *ezrA* locus expressing *ezrA* under the control of P*_spac_*; and replicative pMGPII; Erm^r^, Cm^r^, *lacI*	This study
BCBAJ032	NCTC8325-4Δ*ezrA spa*::P*_spac_ftsZ-cfp*, *lacI*	This study
BCBAJ033	BCBAJ025 (NCTC8325-4 *ezrA::ezrA-mCherry*) with *gfp-pbp2* fusion at the *pbp2* locus, Erm^r^	This study
BCBAJ034	SH1000 with pBCBAJ009 integrated at *ezrA* locus expressing *ezrA* under the control of P*_spac_*; and replicative pMGPII; Erm^r^, Cm^r^, *lacI*	This study
BCBAJ035	Newman with pBCBAJ009 integrated at *ezrA* locus expressing *ezrA* under the control of P*_spac_*; and replicative pMGPII; Erm^r^, Cm^r^, *lacI*	This study
BCBAJ036	BCBAJ001 with pBCBAJ009 integrated at *ezrA* locus expressing *ezrA* under the control of P*_spac_*; *lacI*, Erm^r^, Cm^r^	This study
BCBAJ037	NCTC8325-4 transformed with replicative pBCBAJ003, Erm^r^	This study
BCBAJ038	SH1000 transformed with replicative pBCBAJ003, Erm^r^	This study
BCBAJ039	Newman transformed with replicative pBCBAJ003, Erm^r^	This study
BCBAJ040	SH1000Δ*ezrA*	This study
BCBAJ041	NewmanΔ*ezrA*	This study
BCBAJ042	NCTC8325-4 transformed with pBCBAJ002, Erm^r^	This study
BCBAJ043	NCTC8325-4Δ*ezrA* transformed with replicative pBCBHV003 plasmid; ts, Erm^r^; lacI	This study
BCBAJ044	NCTC8325-4Δ*ezrA spa*:: P*_spac_ -ftsZcfp*	This study
BCBAJ045	NCTC8325-4 transformed with pEPSA5 vector, Cm^r^	This study
BCBAJ046	NCTC8325-4 transformed with pBCBAJ006 expressing *ezrA* antisense RNA under the control of pT5X, Cm^r^	This study
BCBAJ047	SH1000 transformed with pEPSA5 vector, Cm^r^	This study
BCBAJ048	SH1000 transformed with pBCBAJ006 expressing *ezrA* antisense RNA under the control of pT5X, Cm^r^	This study
BCBAJ049	Newman transformed with pEPSA5 vector, Cm^r^	This study
BCBAJ050	Newman transformed with pBCBAJ006 expressing *ezrA* antisense RNA under the control of pT5X, Cm^r^	This study
BCBAJ051	SH1000 transformed with pMGPII which contains *lacI* gene, Cm^r^	This study
BCBAJ052	Newman transformed with pMGPII which contains *lacI* gene, Cm^r^	This study

**Table 3:** Primers used in this study.

Name	Sequence (5′-3′)
EzrAP4	tcagaattcccatatagctgccttgaatg
EzrAP5	ctcgagacttgcagagctagc **tgcagcacttgcaga**ttgcttaataacttcttcttc
EzrAP1	caa**tctgcaagtgctgca** gctagctctgcaagtctcgagaaactagtatgtagttatac
EzrAP2	ccgggatccccattgcaatatcatttggc
mChP3	taagctagcatgattgtgagcaagggcga
mChP4	ttactcgagttacttgtacagctcgtcc
EzrAP22	gccgaattcgataaattaggaggagaagc
EzrAP7	ctgggatcccaagtagacttgctgcctcac
EzrAP8	cgggtacccgatatagcgaggttcagg
EzrAP29	aatttggtgaggcagcaagtct
EzrAP30	gctctaaccttggctcaaatttttc
upEzrAP1	ccggaattcgaagttttcaccgtgtacacc
downEzrAP3	ctacatactagtttctacatatgcttctcctcctaatttatc
upEzrAP2	gataaattaggaggagaagcatatgtagaaactagtatgtag
BTHEzrAP1	ataataggatcctatggtgttatatatcattttggc
BTHEzrAP2	ataatagaattcaattgcttaataacttcttcttca
16SrRNA P3	ggcgaaggcgactttctg
16SrRNA P4	ccacgctttcgcacatcag

restriction sites are underlined; linker is marked in bold.

### General procedures

DNA manipulations and *E. coli* transformations were carried out using standard methods [29]. Restriction enzymes were purchased from New England Biolabs. Polymerase Chain Reaction (PCR) was performed using Phusion high-fidelity DNA polymerase (Finnzymes). Sequencing reactions were carried out at Macrogen.

*S. aureus* RN4220 cells were transformed by electroporation as previously described [30] and plasmids were moved into different *S. aureus* strains by transduction using the phage 80α [31].

### Construction of plasmids and strains

To express *ezrA* from the P*_spac_* inducible promoter, a DNA fragment including the putative RBS and a truncated 5′ region of *ezrA* (533 bp) was amplified by PCR using primers EzrAP22 and EzrAP7. The PCR fragment was digested with EcoRI and BamHI and cloned downstream of the IPTG-inducible P*_spac_* promoter in the pMUTIN4 vector [32], resulting in the plasmid pBCBAJ009. The plasmid was electroporated into strain BCBAJ001 (which carries pMGPII, a multi-copy plasmid encoding an extra *lacI* gene [33]) where it integrated into the *ezrA* chromosomal locus, resulting in strain BCBAJ036. This construct was transduced into the *S. aureus* strains NCTC8325-4, SH1000, Newman and COL, resulting in strains BCBAJ031, BCBAJ034, BCBAJ035 and BCBAJ019, respectively. All procedures were performed in the presence of IPTG 1 mM. Insertion at the correct chromosomal locus was confirmed by PCR.

A second strategy for EzrA depletion in *S. aureus* involved the use of antisense RNA. For that purpose, a 3′end of the *ezrA* gene (566 bp) was amplified from *S. aureus* COL genomic DNA, using primers BTHEzrAP2 and EzrAP8. The resulting PCR product was digested with EcoRI and KpnI and cloned into the pEPSA5 vector [34], resulting in pBCBAJ006, in which the *ezrA* gene fragment is transcribed in the opposite direction compared to the *ezrA* chromosomal copy. This replicative plasmid was introduced into *S. aureus* RN4220 by electroporation and transferred to *S. aureus* strains NCTC8325-4, SH1000, Newman, COL and BCBAJ012 by transduction, selecting with chloramphenicol 20 µg/mL and glucose 0.2% (w/v). Final strains were named BCBAJ006, BCBAJ046, BCBAJ048, BCBAJ050, BCBAJ016 and BCBAJ024, respectively. The empty vector pEPSA5 was also introduced into the same strains which were used as controls and named BCBAJ005, BCBAJ045, BCBAJ047, BCBAJ049, BCBAJ015 and BCBAJ023.

To delete the entire *ezrA* gene from the chromosome of *S. aureus* RN4220 leaving only its start and stop codons (ATGTAA), a 1102 bp fragment upstream of the *ezrA* gene and a 1107 bp fragment downstream of the *ezrA* gene were amplified by PCR from *S. aureus* COL genomic DNA using primer pairs upEzrAP1/downEzrAP3 and upEzrAP2/EzrAP2, respectively. The two fragments were joined by overlap PCR using primers upEzrAP1/EzrAP2, gel purified, digested and cloned into the EcoRI/BamHI sites of pMAD vector, yielding pBCBAJ003. The resulting plasmid was sequenced, introduced into RN4220 cells by electroporation and transduced to *S. aureus* NCTC8325-4, SH1000, Newman and COL using phage 80α. Blue colonies of BCBAJ002, BCBAJ037, BCBAJ038, BCBAJ039 and BCBAJ011 grown at 30°C on TSA with erythromycin and supplemented with X-Gal, were selected. Growth at 43°C with erythromycin allowed selection of colonies in which pBCBAJ003 had integrated at the *ezrA* locus, which was confirmed by PCR. Plasmid excision occurred after a second homologous recombination event, in cells growing in the absence of antibiotic. White, erythromycin sensitive colonies were selected, and *ezrA* gene deletion was confirmed by PCR. The resulting strains were named BCBAJ004, BCBAJ030, BCBAJ040, BCBAJ041 and BCBAJ014.

For localization of EzrA, a C-terminal fusion of mCherry to EzrA was expressed in *S. aureus* from its native locus. For that purpose, two PCR fragments containing the truncated 3′end of *ezrA* gene (1099 bp) without its stop codon and the 1107 bp sequence downstream of *ezrA* gene, were amplified from *S. aureus* COL genomic DNA using primer pairs EzrAP4/EzrAP5 and EzrAP1/EzrAP2, respectively. The two fragments were joined by overlap PCR using primers EzrAP4 and EzrAP2 which resulted in the introduction of a 5 codon linker at the 3′ end of the *ezrA* gene. The resulting fragment was digested with EcoRI/BamHI and cloned into the thermosensitive pMAD vector [35], generating the plasmid pBCBAJ001. The mCherry coding sequence (711 bp) was amplified from the plasmid pBCB4-ChE [24] using primers mChP3 and mChP4 and cloned into pBCBAJ001 using enzymes NheI and XhoI, in frame with *ezrA* gene, generating plasmid pBCBAJ002. This plasmid was introduced into RN4220 cells by electroporation, resulting in strain BCBAJ028, and was transduced from this strain to NCTC8325-4 and COL using phage 80α, generating strains BCBAJ042 and BCBAJ013. These strains were incubated at 43°C in the presence of erythromycin for selection of colonies in which plasmid pBCBAJ002 was integrated into the *ezrA* locus. These colonies were then grown without antibiotic selection. After a second recombination event leading to plasmid excision, cells in which *ezrA* was replaced by an *ezrA-mCherry* fusion were identified by PCR and the resulting strains were named BCBAJ025 and BCBAJ012.

For localization studies of FtsZ we constructed strains where FtsZ-CFP fusion is ectopically expressed, under the control of the P*_spac_* promoter, from the *spa* locus of *S. aureus* chromosome. To construct these strains, phage 80α was used to transduce pBCBHV003 plasmid [36] into the null mutant strain BCBAJ030. The resulting strain BCBAJ043 was incubated at 43°C in the presence of erythromycin to allow the integration of the pBCBHV003 plasmid. Incubation in the absence of antibiotic selection, allowed the selection of white colonies in which the vector had been excised and the *spa* gene was exchanged by *ftsZcfp* generating strain BCBAJ044.

In order to co-localize EzrA and PBP2 in the same cell, a phage lysate of the RNpPBP2-31 strain [37] was used for transduction into BCBAJ025 and BCBAJ012. Colonies expressing GFP-PBP2 were further screened using a 473 nm laser of a FUJI FLA5100 imager and the resulting strains expressing GFP-PBP2 and EzrA-mCherry were named BCBAJ033 and BCBAJ017.

### Growth conditions for EzrA depletion experiments

To evaluate the essentiality of EzrA for *S. aureus* growth, inducible strains BCBAJ036 (RN4220 background), BCBAJ031 (NCTC8325-4 background), BCBAJ034 (SH1000 background), BCBAJ035 (Newman background) and BCBAJ019 (COL background) and the corresponding parental strains containing the plasmid pMGPII, with an extra *lacI* gene BCBAJ001, BCBHV002, BCBAJ051, BCBAJ052 and BCBAJ010, were grown overnight in TSB with erythromycin, chloramphenicol and IPTG 1 mM. The following day, the cultures were diluted 1/200 in TSB supplemented with erythromycin, chloramphenicol and IPTG and grown until mid-exponential phase so that they could recover from stationary phase. At this time, the cultures were washed twice with TSB and diluted in pre-warmed TSB (with or without antibiotics) to an OD_600 nm_ of 0.001. These cultures were split in two and 1 mM IPTG was added to one of the aliquots. To modulate expression of EzrA using antisense RNA, overnight cultures of strains BCBAJ006, BCBAJ046, BCBAJ048, BCBAJ050, BCBAJ016 and BCBAJ024 grown with 20 µg/mL of chloramphenicol and glucose 0.2% (w/v) were diluted 1/500 into TSB (without antibiotics) supplemented with 2% xylose (w/v), to induce the expression of the 3′-end *ezrA* antisense RNA. Control strains transformed with the empty pEPSA5 vector, BCBAJ005, BCBAJ045, BCBAJ047, BCBAJ049, BCBAJ015 and BCBAJ023 were also grown under the same conditions.

To confirm depletion of EzrA upon antisense RNA expression, strain BCBAJ024 was grown in TSB containing 2% xylose (w/v) or 0.2% glucose (w/v) and strain BCBAJ012 was grown in TSB. Cells were harvested from 20 mL of culture, washed with phosphate buffered saline (PBS) and resuspended in 0.2 mL of PBS. Cell lysates were prepared by disrupting cells using a Fast Prep FP120 (Thermo) in 3 cycles of 45 seconds, at maximum speed, with 10 minutes incubation on ice between cycles. Lysates were clarified by centrifugation and 5× SDS-loading buffer (500 mM Dithiothreitol; 10% SDS; 250 mM Tris·HCL, pH 6.8; 30% glycerol; 0.02% bromophenol blue) was added to the supernatant. Samples were heated to 30°C for 10 minutes and analyzed by SDS-PAGE (10%). After running, the gel was washed with MilliQ water and EzrA-mCherry protein fluorescence was detected using a 532 nm laser in a FUJI FLA-5100 reader. The integrated density of the bands was calculated using Image J 1.42q software.

### Quantitative Real-Time PCR

*S. aureus* strains BCBHV002 and BCBAJ030 were grown in TSB until early-exponential phase for RNA extraction. The inducible mutant strain BCBAJ031 was grown with 1 mM IPTG and without IPTG inducer, as indicated above, until early-exponential phase. Prior to harvesting, RNAprotect Bacteria Reagent (twice the culture volume, Qiagen) was added to the culture and the mixture was immediately vortexed for 10 sec. The cells were harvested, the pellet was quickly frozen in liquid N_2_ and stored at −80°C overnight. The next day, the pellet was resuspended with 1 mL of FastRNA blue reagent (MP Biomedicals) and cells were disrupted using silica beads and a FastPrep FP120 apparatus (Thermo). RNA was extracted with 200 µL chloroform and recovered by precipitation with isopropyl alcohol, washed with 80% ethanol, and resuspended in milli-Q water. Integrity of total RNA was analysed in an agarose gel under denaturing conditions (0.25 M formaldehyde) and quantified using a Nanodrop Spectrophotometer ND-100.

cDNA was generated from 300 ng of each RNA sample using TaqMan RT Reagents (Applied Biosystems, Foster City, CA). The reaction mix included 5.5 mM MgCl_2_, 500 µM dNTPs, 2.5 µM random hexamers, 1× RT Buffer, 0.8 U/µl RNase Inhibitor and 1.25 U/µl MultiScribe RT in a final volume of 25 µl. The Reverse Transcription conditions were 10 min at 25°C, 15 min at 42°C and 5 min at 99°C.

Quantification of *ezrA* expression was achieved using the ABI7000SDS (Applied Biosystems), SYBR Green chemistry, and the standard curve method for relative quantification. The PCR reagents consisted of: 1× SYBR Green PCR Master Mix (Applied Biosystems), 400 nM of each primer, and 5 µl of sample cDNA, in a final volume of 25 µl. The thermocycling profile was: 10 min at 95°C followed by 40 cycles of 15 s at 95°C and 1 min at 60°C. *16SrRNA* was used as the endogenous control, and the uniformity of the *16SrRNA* expression levels throughout the heterogeneous samples was confirmed before proceeding with subsequent qPCR comparisons. Briefly, cDNA from all strains subjected to different experimental conditions were generated from exactly the same amount of RNA and were subjected to qPCR analysis as described above. The obtained Threshold cycle (Ct) values varied less than 0.2 between samples from the same strain, and less than 0.7 between samples from different strains (data not shown), unequivocally validating the use of the *16SrRNA* gene as an endogenous control. qPCR primers for both *ezrA* (EzrAP29 and EzrAP30) and *16SrRNA* (16SrRNAP3 and 16SrRNAP4) (Table 3) were designed using ABI7000SDS – specific software, Primer Express (Applied Biosystems).

For each sample, the amount of *ezrA* and *16SrRNA* was determined from the respective standard curve by conversion of the mean threshold cycle values, and normalization was obtained by dividing the quantity of *ezrA* by the quantity of *16SrRNA*. The specificity of the amplified products was verified by analysis of the dissociation curves generated by the ABI 7000 software based on the specific melting temperature for each amplicon.

### Fluorescence Microscopy

*S. aureus* strains were grown overnight in TSB at 37°C with appropriate selection. After reaching the desired OD, 1 ml of cells were pelleted and resuspended in 20 µl of PBS from which 1 µl was placed on a thin layer of 1% (w/v) agarose in PBS, mounted on a slide. Microscopy was performed using a Zeiss Axio Observer.Z1 microscope and images were taken with a Photometrics CoolSNAP HQ2 camera (Roper Scientific) using Metamorph 7.5 software (Molecular Devices).

Labeling of nascent cell wall with Vancomycin-FL was done as previously described [23].

Bocillin-FL (Molecular Probes), a BODIPY-fluorescent derivative of penicillin, was used to visualize PBPs. For that purpose, 1 mL of culture of NCTC8325-4 and BCBAJ030 (NCTC8325-4 *ΔezrA*) cells was incubated with 0.1 µM Bocillin-FL, for 5 minutes, protected from light. Cells were visualized as described above.

### Bacterial Two Hybrid Assays

The *ezrA* gene was amplified from *S. aureus* COL genomic DNA. To construct a fusion of the *ezrA* gene in frame with the 5′ end of the *cya* gene we used primer pair BTHEzrAP1/BTHEzrAP2 and cloned the resulting PCR product into pKNT25 vector [25], resulting in plasmid pBCBAJ010. The *ezrA* gene was also cloned in frame with the 5′end of the *cya* gene into pUT18 vector [25], using primers BTHEzrAP1/BTHEzrAP2, resulting in pBCBAJ011 plasmid. Both constructs were confirmed by sequencing. To test for the existence of putative interactions between EzrA and PBPs, plasmids with the genes coding for each PBP cloned in frame with the *cya* gene were used as previously described [38]. Briefly, all possible combinations of those plasmids were co-transformed into the reporter *E. coli* strain BTH101. The empty vectors pUT18 and pKNT25 were used as negative control, and plasmids p18Zip and p25Zip [25], which express each a domain of a leucine zipper, were used as a positive control.

To qualitatively evaluate putative interactions, cultures of *E. coli* BTH101 cells co-transformed with BTH plasmid pairs were diluted 10^−2^, 10^−4^ to 10^−5^ and 10 µL of each dilution was plated on LA supplemented with X-Gal 40 µg/mL, IPTG 0.5 mM, ampicillin 100 µg/mL and kanamycin 50 µg/mL.

### Electron Microscopy

*S. aureus* strains NCTC8325-4 and BCBAJ030 were grown in TSB until OD600 nm 0.7–0.8. EzrA conditional mutant strain, BCBAJ031, was grown with 1 mM IPTG until mid-exponential phase. The culture was washed twice with TSB, diluted in pre-warmed media to an initial OD_600 nm_ of 0.002 and grown with or without 1 mM IPTG until OD_600 nm_ of 0.8. All samples were washed with 0.1 M sodium-cacodylate buffer (pH 7.4) and fixed with 10 times their pellet volume of ice-cold 2.5% (v/v) glutaraldehyde in 0.1 M sodium-cacodylate buffer, pH 7.4. The cells were then centrifuged, washed with the same buffer, and post fixed with 2% osmium tetroxide for 2 h. After a brief rinse with buffer, the cells were dehydrated using a graded acetone series and embedded in Spurr medium [39]. Thin sections were stained with uranyl acetate and lead citrate and viewed in a Philips BioTwin CM120 electron microscope at 100 kV. Individual micrographs were recorded on Kodak 4489 film at a nominal magnification of 11.000×. At least 2000 cells of each strain were analyzed.

## Supporting Information

Table S1 **Frequency of cells (%) with diameter below and above 1.75 µm for different strains.**
(DOC)
