# Supplementary material for: EzrA Contributes to the Regulation of Cell Size in Staphylococcus aureus
Source: PLoS One. 2011 Nov 14;6(11):e27542. doi: 10.1371/journal.pone.0027542 (PMC3215724; doi:10.1371/journal.pone.0027542)
Supplement: Table S1 — Frequency of cells (%) with diameter below and above 1.75 µm for different strains. (DOC) [file pone.0027542.s001.doc]

**EzrA Contributes to the Regulation of Cell Size in *Staphylococcus aureus***

Ana M. Jorge^1^, Egbert Hoiczyk^2^, João P. Gomes^3^ and Mariana G. Pinho^1,*^

**Supporting Table.**

**Table S1**. Frequency of cells (%) with diameter below and above 1.75µm for different strains.

|  | **Relevant genotype** | **< 1.75 µm** | **> 1.75 µm** | **n^*^** |
| --- | --- | --- | --- | --- |
| **RN4220** | **Wild type** | **84** | **16** | **1032** |
| BCBAJ036 | RN4220 *ezrA*::P*_spac_-ezrA, lacI* | 53 | 47 | 1055 |
| BCBAJ036 with 1mM IPTG | RN4220 *ezrA*::P*_spac_-ezrA, lacI* | 92 | 8 | 1138 |
| BCBAJ004 | RN4220 *ΔezrA* | 44 | 56 | 1026 |
| **NCTC8325-4** | **Wild type** | **93** | **7** | **1028** |
| BCBAJ031 | NCTC8325-4 *ezrA*::P*_spac_-ezrA, lacI* | 28 | 72 | 887 |
| BCBAJ031 with 1mM IPTG | NCTC8325-4 *ezrA*::P*_spac_-ezrA, lacI* | 52 | 48 | 1026 |
| BCBAJ030 | NCTC8325-4 *ΔezrA* | 46 | 54 | 1092 |
| **SH1000** | **Wild type** | **98** | **2** | **1242** |
| BCBAJ034 | SH1000 *ezrA*::P*_spac_-ezrA, lacI* | 55 | 45 | 1030 |
| BCBAJ034 with 1mM IPTG | SH1000 *ezrA*::P*_spac_-ezrA, lacI* | 84 | 16 | 1021 |
|  |  | **< 1.5 µm^+^** | **> 1.5 µm^+^** |  |
| **COL** | **Wild type** | **98,7** | **1,3** | **955** |
| BCBAJ019 | COL *ezrA*::P*_spac_-ezrA, lacI* | 59,3 | 41,9 | 680 |
| BCBAJ019 with 1mM IPTG | COL *ezrA*::P*_spac_-ezrA, lacI* | 87,2 | 12,8 | 920 |
| BCBAJ014 | COL *ΔezrA* | 45 | 55 | 1708 |
|  |  |  |  |  |

*Total number of cells quantified (n).

+1.5 µm threshold was used for strain COL due to the smaller size of cells from this strains.
